# Traffic Signal Timing Optimization Model Based on Video Surveillance Data and Snake Optimization Algorithm

**DOI:** 10.3390/s23115157

**Published:** 2023-05-28

**Authors:** Ruixiang Cheng, Zhihao Qiao, Jiarui Li, Jiejun Huang

**Affiliations:** School of Resources and Environmental Engineering, Wuhan University of Technology, Wuhan 430070, China; 315843@whut.edu.cn (R.C.); 315877@whut.edu.cn (Z.Q.); 315865@whut.edu.cn (J.L.)

**Keywords:** signal timing optimization, simulation, snake optimization algorithm, traffic congestion

## Abstract

With the continued rapid growth of urban areas, problems such as traffic congestion and environmental pollution have become increasingly common. Alleviating these problems involves addressing signal timing optimization and control, which are critical components of urban traffic management. In this paper, a VISSIM simulation-based traffic signal timing optimization model is proposed with the aim of addressing these urban traffic congestion issues. The proposed model uses the YOLO-X model to obtain road information from video surveillance data and predicts future traffic flow using the long short-term memory (LSTM) model. The model was optimized using the snake optimization (SO) algorithm. The effectiveness of the model was verified by applying this method through an empirical example, which shows that the model can provide an improved signal timing scheme compared to the fixed timing scheme, with a decrease of 23.34% in the current period. This study provides a feasible approach for the research of signal timing optimization processes.

## 1. Introduction

The ascendancy of urbanization and inadequate public transportation amenities have fostered a consistent upsurge in the worldwide possession of private automobiles [1,2]. While conferring convenience, the increase in the number of cars has concurrently engendered profound traffic congestion, thereby facilitating peril in terms of traffic safety, ecological contamination, and substantial economic ramifications. These hazards have impeded both societal and economic progress, thus becoming a constraining bottleneck that curtails the steady and harmonious evolution of urban centers [3]. In response to the global push for carbon neutrality and carbon peaking, China has been actively exploring theories and methods to alleviate traffic congestion. One topic that has attracted considerable academic attention is the role of intersection traffic signals in mitigating congestion by regulating the behavior of vehicles [4]. Suitable timing schemes can greatly alleviate traffic congestion, and studies have found that increasing road traffic efficiency, shortening vehicle waiting time, and reducing pollution emissions are important indicators for quantifying the suitability of a timing scheme [5].

The conventional timing algorithms can be broadly classified into three categories. The first category is based on traditional mathematical modeling methods, which include fuzzy logic and graph theoretic modeling algorithms [6]. Fuzzy logic algorithms employ timing control through the use of a fuzzy logic controller (FLC) to compare different proposed types of traffic light control systems based on waiting time and queue length [7]. Graph theoretic modeling algorithms transform intersection problems into a linear programming problem (LP) with the aim of minimizing waiting time by representing intersections as a network [8]. The second category comprises intelligent optimization algorithms, which are based on iterative population intelligence, such as genetic algorithms, in addition to simulated annealing algorithms and ant colony algorithms [9]. The third category is based on deep learning methods, such as reinforcement learning [10]. Moreover, time difference-based methods, along with Markov games, can generate reinforcement learning models [11]. At present, the excellent results produced by research on the convergence of different methods are encouraging. The combination of classical queuing and scheduling theory with reinforcement learning increases the efficiency of the timing strategy [12], and the network modeling approach is used for the extraction of key nodes in a way that is incorporated into the deep learning model [13]. Most of these studies have produced a multitude of software system outcomes, and the utilization of multiple algorithms contributes to the complexity of these systems. Therefore, it is crucial to employ an appropriate framework to ensure the accuracy of complex systems. Furthermore, there are already relevant studies utilizing machine learning approaches to assess the rationality of such complex systems [14,15].

This study focuses on detection timing for the current period [16,17], while the resulting scheme is applied in the next period. It is challenging to simultaneously incorporate traffic flow monitoring and traffic flow prediction. Therefore, as part of this study, a comprehensive solution is proposed for intersections based on the utilization of video surveillance data, specifically for the real-time monitoring of traffic flow at intersections. Road traffic flow information is first obtained through this process. Subsequently, the LSTM model predicts traffic flow for the next period. Finally, the predicted traffic flow data are fed to VISSIM for simulation, and the timing scheme is optimized using the SO algorithm. This allows for the establishment of a set of intersection timing models, which can serve as a valuable reference for future intelligent traffic systems.

The remainder of the paper is structured as follows. Section 2 introduces the related works of signal timing and the application of the SO algorithm. Section 3 introduces the three parts of algorithmic methods for model detection, prediction, and optimization. Section 4 presents the results and performance of the algorithm and analyzes the results. Finally, the conclusion and further research directions are presented in Section 5.

## 2. Literature Review

In terms of their application, timing methods are typically tested through simulation software due to the complexity and variability of realistic traffic flows [18]. Among these simulation software, VISSIM 4.30 is widely used as a powerful traffic simulation software [19,20]. In the field of timing optimization simulation, the primary emphasis is typically placed on the timing of current road conditions. However, these schemes may not remain applicable in the subsequent periods, presenting a challenge for predicting road conditions and timing adjustments for future periods based solely on the detection of current road conditions. Hence, this poses a challenge for existing signal timing schemes to accurately predict road conditions and to determine suitable timings for subsequent periods based solely on the detection of current road conditions.

The optimization of signal timing through multi-intelligence simulation techniques and the use of Synchro confirm the benefits of simulation software in achieving efficient traffic flows with different vehicle priorities [21]. Nonetheless, the current methods are limited to existing datasets and have therefore demonstrated limited usefulness in practical scenarios. Consequently, the mainstream direction consists of using video detection to gather traffic flow and lane information on driving sections for timing model data. Compared with previous sensors and microwave radar, video surveillance offers advantages such as wide detection range and visually confirmed results [22,23]. Additionally, the inherent capture system of violation photography can reduce monitoring costs, and using video surveillance to detect traffic flow parameters has become a popular topic [24]. The combination of timing models based on video surveillance data and deep reinforcement learning constitute one of the typical applications [25,26]. The use of video surveillance data helps generate accurate predictions of traffic flow, which subsequently enables time-sensitive optimization solutions. The focus of the current study is on the unification of traffic flow prediction and signal timing problems while verifying their effectiveness in public datasets and pursuing the integrity of the entire timing process.

Heuristic algorithms are the existing techniques used to solve signal timing optimization problems. The typical intelligent algorithms, such as genetic [27] and particle swarm optimization [28] algorithms, all have some drawbacks, such as ease of falling into a local optimum and slow operation efficiency. Thus far, many new intelligent optimization algorithms have been proposed to solve these problems. Among them, the snake optimization algorithm has been applied in the fields of engineering optimization and image segmentation, among others [29,30], due to its efficient solution efficiency and fast iteration speed. Moreover, the snake optimization algorithm has shown very good results in coupling work with other algorithms, such as noise denoising, gas outburst prediction, etc. [31,32].

## 3. Materials and Methods

### 3.1. Model Building

For optimal matching system exploration in this study, mainstream video monitoring data are used for traffic monitoring. The YOLOX model is used to detect incoming and outgoing vehicles and to record hourly traffic flow. For the prediction component, the LSTM model is used, with training data consisting of historical data and detected traffic flow detection data as a supplemental input to predict the traffic flow for the next period. Finally, the detected data are used as input to VISSIM for simulation.

**Figure 1:**
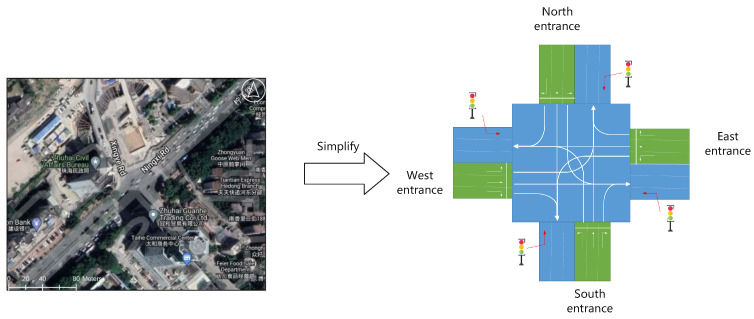
Schematic figure of the simplified road section model.

Based on the analysis of the problem, the intersection can be simplified as shown in Figure 1.

To ensure clear understanding and avoid confusion throughout the research project, it is important to establish definitions for specific symbols beforehand. As outlined in Table 1, the agreed upon meaning of each symbol is the default interpretation unless explicitly stated in subsequent discussions.

### 3.2. YOLOX-Based Video Monitoring of Traffic Flow

Before intelligently regulating traffic signals, it is necessary to partition the road lanes and to perform vehicle target detection for each lane, allowing for traffic flow counting on the road section. To accommodate multiple lanes in the same direction, they can be intelligently set up by VISSIM [33]. Hence, only the lanes separating vehicle travel directions require partitions.

To partition the lanes, the video frames should first be converted to grayscale pictures, and Gaussian filtering should be applied to reduce noise and to blur the pictures. Representing the picture grid matrix as $Q_{mn}$ with the sum of the grid values as *I* and the new picture as *I*${}^{\prime }$, Gaussian transformation is given by:

(1) $$I_{ij}^{\prime }=\frac{1}{S}\sum_{m=i-2}^{i+2}\sum_{n=j-2}^{j+2}Q_{mn}I_{mn}.$$

**Figure 2:**
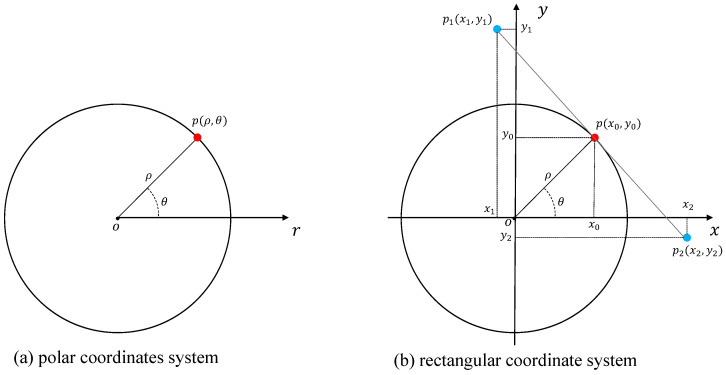
Hough test result conversion schematic.

Once the transformation is completed, the Canny edge detection technique [34] is used to identify road object edges. The detected edges are then subjected to straight line detection using the Hough transform algorithm [35] to extract lane lines. The Hough transform algorithm operates on the point-line duality principle with the conversion process illustrated in Figure 2.

In Figure 2a, the Hough transform result is expressed in polar coordinates as p(ρ,θ). In Figure 2b, the polar coordinate point is expressed in the Cartesian coordinate system as $p(x_{0},y_{0})$, the Hough transform result corresponds to the two endpoints of the line $p_{1}\left(x_{1},y_{1}\right)$ and as $p_{2}\left(x_{2},y_{2}\right)$, the scale factors λ and η are introduced to determine the position of the endpoints $p_{1}$ and $p_{2}$ of the line, and the transformation equation is:

(2) $$\left\{\begin{array}{c} x_{1}=\left[x_{0}-\lambda \sin\left(\theta \right)\right]\;\\ x_{2}=\left[x_{0}+\eta \sin\left(\theta \right)\right]\;\\ y_{1}=\left[y_{0}+\lambda \cos\left(\theta \right)\right]\;\\ y_{2}=\left[y_{0}-\eta \cos\left(\theta \right)\right] \end{array}\right.,\;x_{0}=\rho \cos\left(\theta \right),y_{0}=\rho \sin\left(\theta \right)$$

For vehicle detection, the YOLOX algorithm [36], which is more efficient, is used in this study due to the rapid change in traffic vehicles; thus, the choice of YOLOX-s helps in solving the vehicle target detection problem.

The YOLOX-s algorithm consists of three components: backbone network, data enhancement processing, and prediction:

(1) For the backbone network, the CSPDarknet network is used for feature extraction [37].
(2) For data enhancement, upsampling and feature fusion are performed using three effective feature layers extracted from the backbone network. After the feature fusion process, the results are stacked, and downsampling and further feature stacking procedures are carried out.
(3) For prediction, the YOLOHead is used, which utilizes convolutional normalization and SiLU activation functions for feature integration to obtain confidence levels, regression coefficients, and objects.

**Figure 3:**
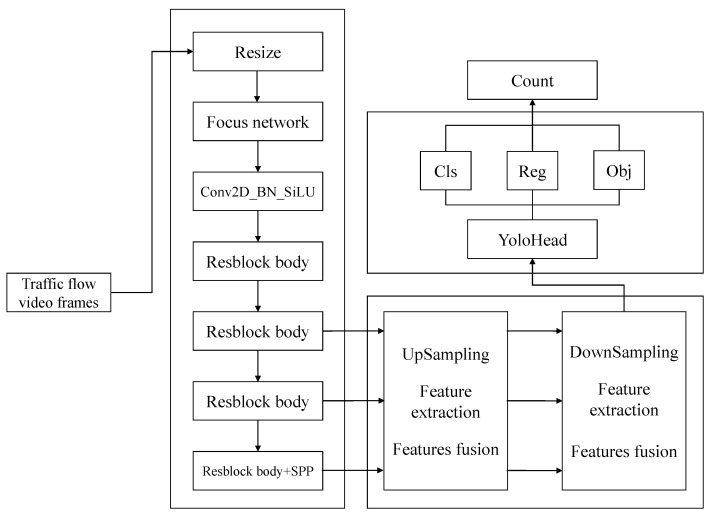
YOLOX structure schematic.

In this way, the structure of YOLOX is constructed, as shown in Figure 3:

### 3.3. LSTM-Based Traffic Prediction

The detection section acquires traffic flow data from the traffic monitoring section. Pre-processing of these data is necessary before employing the LSTM model on historical data. The results obtained from the LSTM model are then integrated with the detected traffic flow data to predict the respective flows for the next period. Data pre-processing can be accomplished by dividing the dataset and by implementing a sliding window approach [38].

**Figure 4:**
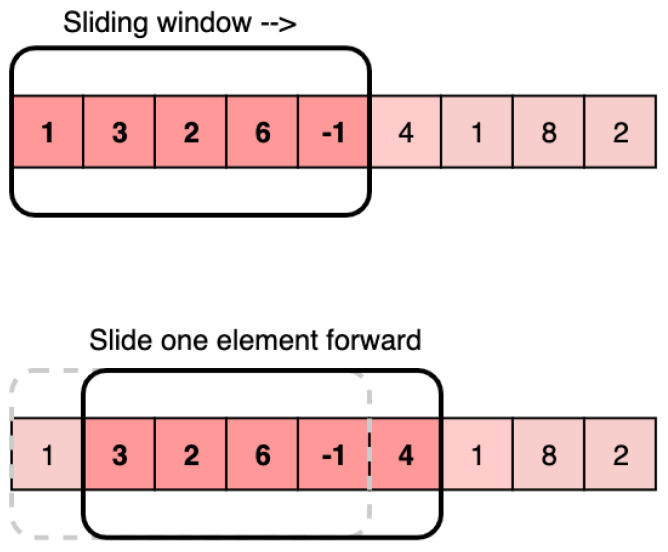
Sliding window schematic.

In real-world traffic scenarios, people tend to travel during peak times, resulting in time sequence data with distinct starting points and ending points. Given the nature of such time sequence data, it becomes crucial to prioritize recent data when predicting traffic flow. Therefore, in the process of data inflow, the sliding window can dynamically update the data in the window in real time by synchronously changing the start and end times of the window [39] to meet the real-time demand and to reduce error for the subsequent traffic simulation. Figure 4 is a schematic of the sliding window.

**Figure 5:**
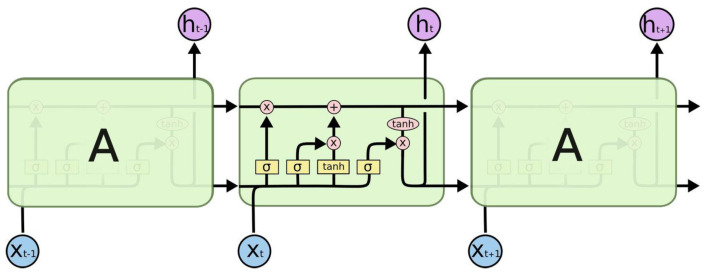
Predictive model schematic. “A” represents LSTM Cell which is a hidden layer neuron.

The LSTM model is an advanced version of the recurrent neural network that can overcome the limitation of RNNs in handling long-term dependencies [40]. In addition, this model can capture both short- and long-term temporal dependencies as well as improve prediction performance by leveraging missing patterns. Figure 5 is a diagram of the LSTM model construction process.

The LSTM algorithm consists of four main components: input gates, forgetting gates, cell states, and output gates.

(1) The input gate $i_{t}$ is used to update the cell state by passing the previous layer of hidden state information with the current input information to the next layer to determine the importance of the updated data via the following equation:
  (3) $$i_{t}=\sigma \left(W_{ii}x_{t}+b_{ii}+W_{hi}h_{t-1}+b_{hi}\right)$$
(2) The forget gate $f_{t}$ decides to discard or retain information from the previous hidden state, and the current input information is passed to the next layer simultaneously after the sigmoid function with the following equation:
  (4) $$f_{t}=\sigma \left(W_{if}x_{t}+b_{if}+W_{hf}h_{t-1}+b_{hf}\right)$$
(3) The cell state $g_{t}$ passes the previous layer with the current input information to the tanh function to create a candidate vector g, which is formulated as follows:
  (5) $$g_{t}=\tanh\left(W_{ig}x_{t}+b_{ig}+W_{hg}h_{t-1}+b_{hg}\right)$$
(4) The output gate $o_{t}$ is used to determine the next hidden state value by passing the previous input information into the sigmoid function to obtain the output value, which is given by the following equation:
  (6) $$o_{t}=\sigma \left(W_{io}x_{t}+b_{io}+W_{ho}h_{t-1}+b_{ho}\right)$$

The LSTM model has two custom parameters $h_{0}$ and $c_{0}$, which are the initial hidden state and the initial cell state, respectively. h(t) and c(t) of the next state are obtained by calculation. h(t) has more memory of new information and changes faster as *t* changes; c(t) records more and earlier information and changes more slowly than *t*. They are calculated as below:

(7) $$\begin{array}{c} c_{t}=f_{t}⊙c_{t-1}+i_{t}⊙g_{t}\\ h_{t}=o_{t}⊙\tanh\left(c_{t}\right) \end{array}$$

where ⊙ is the Hadamard product, which represents the multiplication of the corresponding elements of the matrix.

For the LSTM model used in this paper, the number of datum passed to the program for training is set to 1, the size of the input data is the length of the training data, the size of the hidden state hidden_size is set to 20, the hidden layer layers_size is set to 3, and the number of iterations is 300.

### 3.4. Timing Optimization Model Based on VISSIM Simulation

**Figure 6:**
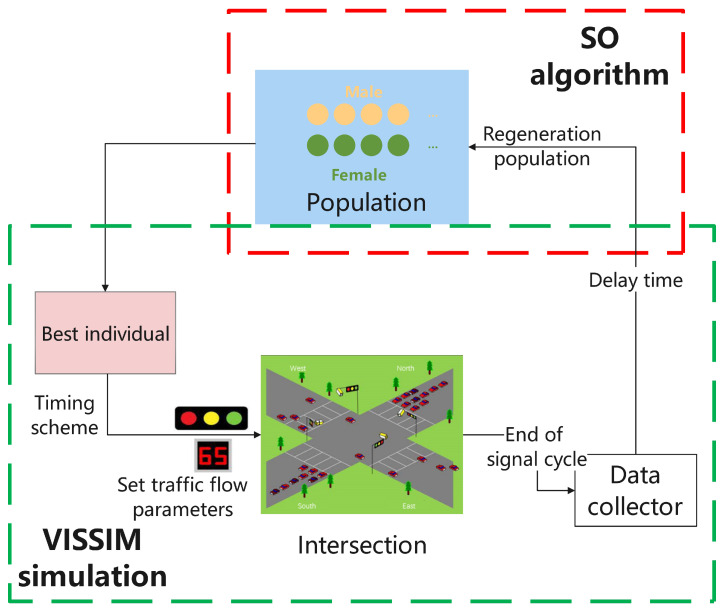
Timing model figure.

Obtaining the necessary input data for the timing model involves detection and prediction. The input data are then integrated into VISSIM simulation software, which is operated by Python. During the optimization process, VISSIM returns key evaluation indices, including delay time and queue length, to the model. Finally, the parameters that control the timing model are optimized using the SO intelligent optimization technique. A schematic illustration of the process is presented in Figure 6.

In traffic signal control, time delay has a significant impact on the evaluation of current traffic flow and is therefore often used as a key indicator of traffic effectiveness. The Webster signal cross delay formula, which is widely used in this area, calculates the delay as follows:

(8) $$D_{i}=\frac{c{\left(1-g_{i}\right)}^{2}}{2\left(1-y_{i}\right)}+\frac{y_{i}^{2}}{2q_{i}g_{i}\left(g_{i}-y_{i}\right)},$$

where *c* is the time of one cycle, $g_{i}$ is the green signal ratio, $q_{i}$ is the corresponding traffic flow of phase *i*, and $y_{i}$ is the saturation of phase *i*.

Since the above equation is valid only when the saturation is low, Yang [41] improved it using the following equation:

(9) $$D_{i}=\frac{cq_{i}{\left(x-y_{i}\right)}^{2}}{2x^{2}\left(1-y_{i}\right)}+\frac{x^{2}}{2(1-x)}$$

where *x* is saturation of the intersection.

In order to have a reasonable criterion for the timing scheme, the mainstream average vehicle delay time of the cycle is used as the evaluation index for evaluation of the signal timing scheme; thus, minimizing the formula is the objective of the optimization function:

(10) $$\min\;D=\frac{1}{n}\sum_{i=1}^{n}\frac{D_{i}}{N_{i}}$$

where *n* is the number of lanes, $D_{i}$ is the cycle delay time of the *i*-th lane, $N_{i}$ is the cycle flow of each lane, and *D* is the average cycle vehicle delay time.

During each traffic cycle, specific durations are allocated to allow for traffic flow in different directions. The green light duration should not exceed 70 s, with the main flow of traffic allocated a minimum of 15 s and the secondary flow allocated a minimum of 8 s. The yellow light duration is typically set to 3 s. In the non-saturated traffic state, the red light duration should not exceed 120 s, while in the saturated traffic state, the combined red light duration should not exceed 150 s. The specific situation in the study area is 170 s in one cycle; thus, only green light timing is considered with the following constraints:

(11) $$\begin{array}{c} t_{g}+t_{r}+t_{y}=T,\;t_{g}\in \left[35,55\right] \end{array}$$

where $t_{g}$ represents the green light time in one cycle, $t_{r}$ represents the red light time in one cycle, $t_{y}$ represents the yellow light time in one cycle, which is 3 s, and *T* represents the running time in one cycle, which is 170 s.

In practice, timing optimization cannot usually be directly applied. Therefore, VISSIM simulation software is commonly employed to perform simulations. Due to the inherent randomness of the simulation process, for each evaluation of a timing scheme, running of 10 cycles is required to obtain the average delay time with a fixed random seed. During system operation, traffic flow values are recorded at hourly intervals to optimize timing. The parameters that define the road network are specified in Table 2 [24].

## 4. Results and Discussion

### 4.1. Traffic Flow Detection Result

**Figure 7:**
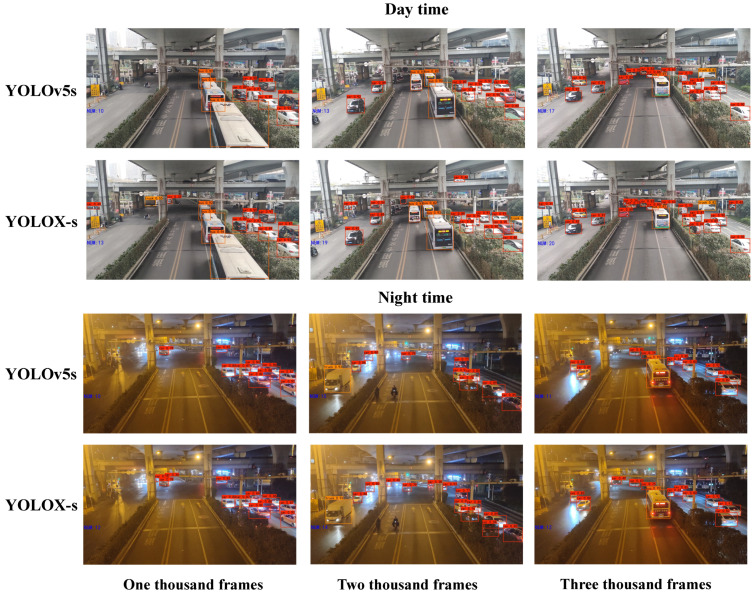
YOLOX detection results.

In this paper, the YOLOX-s model is used, with YOLOv5s [42] included for comparison as shown in Table 3, and the official model file can be directly used for vehicle detection, since it is only for vehicle detection, and setting the conf threshold to 0.3 and nms threshold to 0.3, the prediction results of 1000, 2000, and 3000 frames of daytime and nighttime traffic flow video are intercepted, as shown in Figure 7.

Table 3 reveals the results of three vehicle detection experiments using YOLO series algorithms. YOLOX outperforms YOLOv3-v5 in detecting cars, trucks, and buses, with significantly higher accuracy for cars (83.12%) compared to YOLOv5, resulting in a 5.81% improvement. Additionally, YOLOX has a higher frame rate (77 FPS), highlighting its effectiveness in both identifying and detecting vehicles in real time. These findings demonstrate the validity of selecting YOLOX for vehicle detection.

In Figure 7, YOLOX-s can still detect all vehicles in a single two-way lane at night and distinguish trucks and cars in the frame 2000 time zone and buses and cars in the frame 3000 time zone. In addition, YOLOX-s detects more vehicles than YOLOv5s. YOLOX-s can detect some vehicles at a relatively long distance or that are partially blocked by ground objects at intersections. In daytime vehicle detection, YOLOX-s has more obvious advantages due to better lighting conditions, and more vehicles are detected.

Compared with YOLOv5s, YOLOx-s improves the coupling head and designs it as a multi-branch structure, in which 1×1 convolution is used for dimension reduction. This design is more conducive to the recognition of occluded ground objects. Additionally, YOLOx-s employs multi-positive normality, in which the location near the object center is included in the calculation of positive samples. SimOTA optimal transmission is also utilized to enhance the screening of preselection frames, benefiting the recognition of smaller ground objects. Furthermore, YOLOx-s enhances the loss function by using the IOU loss function to train the regression branch and the BCE loss function to train the classification and objectness branches, improving the accuracy of the model. These innovations form a solid foundation for traffic counting and VISSIM simulation to regulate signals.

### 4.2. Traffic Forecast Results

**Figure 8:**
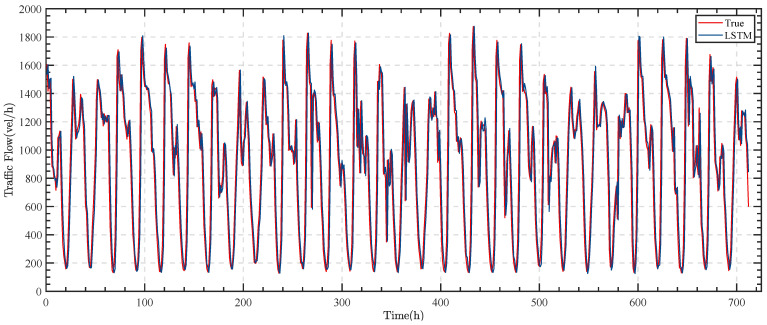
The prediction effect of the LSTM model.

The LSTM model is applied to make predictions, and the results are shown in Figure 8.

As can be seen in Figure 8, the overall fit of the LSTM model matches the actual one, but there is a significant lag, which is usually close to the actual value of traffic flow at time t at moment t + 1.

**Figure 9:**
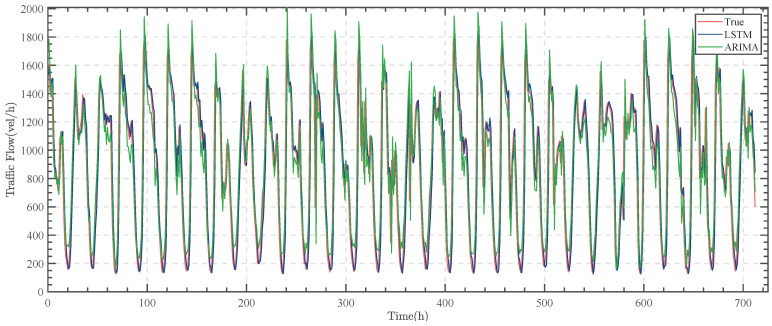
Comparison of the prediction effect of the ARIMA model and LSTM model.

To evaluate the effectiveness of the LSTM, the ARIMA model [43] is also utilized for comparison. The results are shown in Figure 9.

When the data exhibit minimal fluctuation, the ARIMA model tends to perform better than the LSTM model. However, with a larger range of variation, particularly in the traffic monitoring dataset, the LSTM model is more suitable. These findings suggest that while the ARIMA model may be preferable under certain circumstances, the LSTM model is more effective when dealing with highly variable data.

This paper utilizes the mean squared error (MSE) metric to evaluate the effectiveness of the model. The ARIMA model produces an MSE of 446,844.67, while the LSTM model generates an MSE of 54,205.44. The significantly lower MSE of the LSTM model demonstrates its superior predictive capability, particularly in traffic flow prediction. Therefore, the LSTM model is more appropriate for this dataset.

### 4.3. Results of Timing Optimization Model

#### 4.3.1. Model Solving and Algorithm Comparison

Following the construction of the simulation environment, the optimal timing scheme is determined through the application of the snake optimization (SO) algorithm. Developed by Hashim [44] et al. in 2022, this intelligent optimization algorithm has attracted significant attention from practitioners in the optimization industry due to its remarkable convergence speed, minimal parameters, and superior accuracy. Hence, the use of SO is appropriate at this stage.

This algorithm divides the merit-seeking population into male and female groups to achieve merit-seeking goals via exploration, mating, and competition. The weaker solutions are gradually eliminated, which enables optimization of the final results. The parameters are set in Table 4.

In Table 4, num represents the number of iterations, pop is the populations, $C_{f}$ presents the food threshold, $C_{t}$ is the temperature threshold for mating, $C_{1}$ represents the food quality constant, $C_{2}$ represents the update position, and $C_{3}$ is for the constant for fighting and mating.

Given the stochastic nature of intelligent optimization algorithms, the procedure is executed multiple times to ensure selection of the optimal result. Table 5 presents the corresponding outcomes of the optimal SO algorithm and fixed matching time before optimization.

The reduction of 23.34% in the final delay time after optimization when compared to the fixed timing scheme is indicative of a more substantial optimization effect. This outcome highlights the efficacy of the algorithm in addressing the problem at hand.

The following diagram illustrates the comparison between the iteration diagram of the algorithm and those of the genetic algorithm (GA) and particle swarm optimization algorithm (PSO).

**Figure 10:**
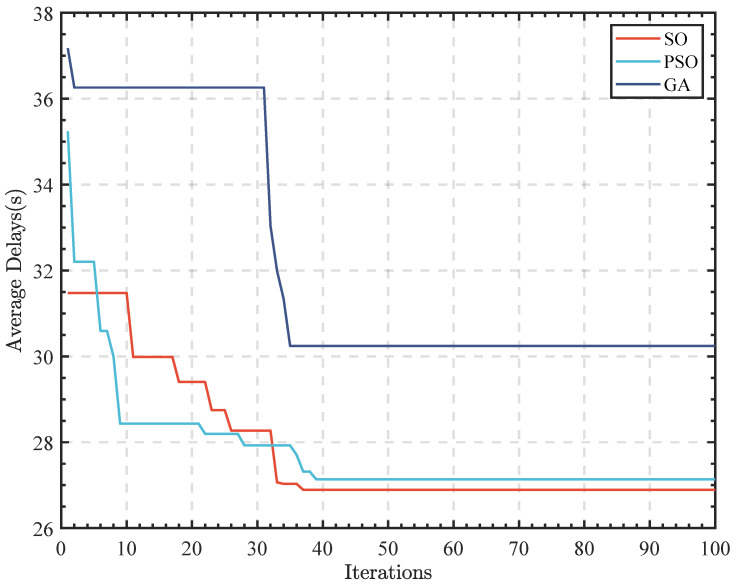
SO, PSO, and GA algorithm iteration.

In Figure 10 and Table 6, it is shown that the algorithm reaches convergence at about 40 iterations. The comparison of traditional GA and PSO shows that the SO iteration converges fastest and can achieve lower average delay speed, thus highlighting the superiority of the SO algorithm in solving this problem.

#### 4.3.2. Algorithm Time Spend

In practical applications, the time consumed by the timing process is crucial for the system to generate optimal signal timing schemes considering the current road conditions in real time. The detection and prediction models exhibit higher efficiency and require less time compared to the optimization model. Hence, it is necessary to evaluate and discuss the operational efficiency of the detection and prediction models, as their performance significantly impacts the overall efficiency of the system.

The average running times of the GA, PSO, and SO algorithms are shown in Table 7.

As can be seen from Table 7, the SO algorithm significantly outperforms the GA and PSO algorithms in terms of running time. It is also sufficient in terms of time spent to provide a timing solution for the next period, since the optimization model section gives a timing solution for the next 1 h.

#### 4.3.3. Model Sensitivity Analysis

Traffic flow is a very important parameter in the timing model that can have a great impact on the overall timing results. However, the predicted and actual traffic flow are hardly consistent, and the prediction error will inevitably produce some fluctuations in the final timing results. In order to test the impact of the prediction accuracy on the timing results of the SO algorithm optimization, we fluctuated the corresponding traffic volume by 1%, 2%, and 5%, and the final results are shown in Table 8.

When there is a change in traffic volume to small, the model results change relatively very little, while when the traffic volume changes to large, there is a relatively greater change in results. However, the overall change in the results is still smaller compared to the degree of parameter change, indicating that the timing model can be considered stable.

## 5. Conclusions

This study aimed to investigate the signal timing problem to reduce traffic congestion and to enhance road traffic flow, thereby supporting sustainable urban development. Rather than conducting a comprehensive study of the overall timing scheme and process, the focus of the current research remained on observing and predicting the timing process or merely monitoring timing as in most prior research. In this study, we take a different approach by integrating traffic flow video monitoring, traffic flow period prediction, and optimization of the next period timing scheme. This interconnected approach allows for a more comprehensive analysis of the signal timing problem. Furthermore, we assess the effectiveness of emerging optimization algorithms, such as SO, in comparison to traditional counterparts. Based on the previous results, we draw the following conclusions:

(1) The YOLOX model has strong capability for road vehicle detection. Under the premise of dividing lanes, the vehicles of each different lane are counted, and the traffic flow of each road is determined on an hourly basis. The results show that the YOLOX model can still perform the function of vehicle detection well even under complicated road conditions with complete counting statistics of road vehicles.
(2) The LSTM model is able to perform the forecasting task better than in the traffic forecasting module. The LSTM model used in the forecasting module is transformed into a sliding window model for traffic forecasting to predict the effect of the next period, which greatly improves the forecasting effect. This also highlights the accuracy of the prediction results compared to the traditional time-series prediction model ARIMA.
(3) The timing optimization model can provide better timing solutions. After establishing the optimization objective, the information of each traffic flow obtained by VISSIM is used as the basis for the iterative update of the algorithm, and after optimization of the model using the SO algorithm, the results show that the delay time is reduced by 23.34% compared with traditional fixed timing. The algorithm is also compared with the traditional intelligent optimization algorithms GA and PSO in highlighting the superiority of the algorithm.

This paper synthesizes the main research directions of the current signal timing model to unify the whole process. However, it is also necessary to consider not only vehicle traffic flow but also the impact of human flow, special lanes, and traffic flow of other nearby intersections on the signal timing results for the current intersection. This consideration is crucial given the complexity and uncertainty of real traffic flow.

## Figures and Tables

**Table 1:** Related parameters.

Symbol	Description	Unit
*D*	Average delay time of vehicles in one cycle	s
*T*	Duration of one phase cycle	s
$q_{i}$	Phase *i* traffic flow (vel/h)	(vel/h)
$t_{g}/t_{r}/t_{y}$	Duration of green/red/yellow lights in one cycle	s
*Q*	Video frame grayscale matrix	
$c_{i}$	The cell state at each stage at moment t	
$h_{i}$	The hidden state at time t	
*I*	Image grayscale matrix value	
*S*	Sum of grayscale values	
*n*	Number of lanes	
$N_{i}$	Flow rate for each lane in one cycle	
$y_{i}$	Phase *i* corresponds to the road saturation	

**Table 2:** Simulation parameter settings.

Item	Properties
Number of Lanes	Bidirectional, six lanes only controlling left turn and straight
Lane Width (m)	3.5
Lane Length (m)	400
East–West Flow (veh/h)	2376
North–South Flow (veh/h)	2950
Vehicle Type Ratios	Cars:Buses:Trucks = 0.9:0.08:0.02
Desired Speed (km/h)	Cars:Buses:Trucks = 50:40:30

**Table 3:** YOLO series vehicle detection effect comparison.

Network Model	AP@0.5 (%)			Recall	mAP (%)	FPS/(f/s)
	Car	Bus	Truck			
YOLOv3	68.32	57.83	58.46	55.26	61.54	27
YOLOv4	72.23	60.78	61.94	59.08	64.98	48
YOLOv5s	77.31	62.67	63.08	61.73	67.69	69
YOLOX-s	83.12	64.43	65.26	63.44	70.94	77

**Table 4:** Parameter settings for SO algorithm.

num	pop	$C_{f}$	$C_{t}$	$C_{1}$	$C_{2}$	$C_{3}$
100	10	0.25	0.6	0.5	0.5	2

**Table 5:** Comparison of SO algorithm results with fixed time allocation scheme.

Phases	SO Optimization Solution			Fixed Timing Solutions		
	Red Light (s)	Green Light (s)	Yellow Light (s)	Red Light (s)	Green Light (s)	Yellow Light (s)
N Straight, N Left	126	41	3	120	47	3
S straight, S left	128	39	3	112	55	3
NE straight, NE left	120	47	3	114	53	3
SW straight, SW left	120	47	3	114	53	3
Avg. delay time (s)	26.89			35.08		

N for north, NE for northeast, S for south, SW for southwest.

**Table 6:** Average delay time when algorithm converges.

Algorithm	GA	PSO	SO
Avg. delay time (s)	30.46	27.21	26.89
Less than fixed solutions (%)	13.17	22.34	23.34

**Table 7:** Comparison of time consumption by algorithms.

Algorithm	GA	PSO	SO
Runtime (s)	33.5	17.8	12.7

**Table 8:** Ratio of original vehicle flow.

Ratio to Original Vehicle Flow	0.95	0.98	1	1.02	1.05
Optimized delay time (s)	26.72	26.76	26.89	27.35	27.89
Ratio of change to original result	0.63%	0.48%	0.00%	1.71%	3.72%

## Data Availability

Not applicable.
